# Impaired fibrinolysis in patients with atrial fibrillation and elevated circulating lipopolysaccharide

**DOI:** 10.1007/s11239-024-02980-5

**Published:** 2024-04-21

**Authors:** Marcin Sadowski, Michał Ząbczyk, Anetta Undas

**Affiliations:** 1 Collegium Medicum, Jan Kochanowski University, Kielce, Poland; 2 Department of Thromboembolic Disorders, Institute of Cardiology, Jagiellonian University Medical College, Krakow, Poland; 3 Krakow Centre for Medical Research and Technologies, John Paul II Hospital, Krakow, Poland

**Keywords:** Lipopolysaccharide, Gut microbiota, Clot properties, Atrial fibrillation, Fibrinolysis

## Abstract

It is unknown whether elevated gut-derived serum lipopolysaccharide (LPS) can affect thrombin generation, fibrinolysis, and fibrin clot properties in atrial fibrillation (AF). We aimed to evaluate associations of circulating LPS with prothrombotic markers in AF patients. A total of 157 (women, 57.3%) ambulatory anticoagulant-naïve AF patients aged from 42 to 86 years were recruited. Clinical data together with serum LPS, inflammation, endothelial injury, coagulation and fibrinolysis markers, including fibrin clot permeability (K_s_) and clot lysis time (CLT), were analyzed. A median LPS concentration was 73.0 (58.0-100.0) pg/mL and it showed association with CLT (*r* = 0.31, *p* < 0.001) and plasminogen activator inhibitor-1 (PAI-1, *r* = 0.57, *p* < 0.001), but not other fibrinolysis proteins, thrombin generation, inflammatory markers, or K_s_. There were weak associations of LPS with von Willebrand factor (vWF, *r* = 0.2, *p* = 0.013), cardiac troponin I (*r* = 0.16, *p* = 0.045), and growth differentiation factor-15 (*r* = 0.27, *p* < 0.001). No associations of LPS and CHA_2_DS_2_-VASc or other clinical variables were observed. Multivariable regression adjusted for potential confounders showed that serum LPS ≥ 100 pg/mL was an independent predictor of prolonged CLT. This study is the first to demonstrate antifibrinolytic effects of elevated LPS in AF patients largely driven by enhanced PAI-1 release.

## Keypoints

Lipopolysaccharide occurs in blood as a result of the intestinal barrier breakdown.

Low-grade gut-derived endotoxemia contributes to a prothrombotic clot phenotype.

Antifibrinolytic effect is driven by plasminogen activator inhibitor-1 release.

Potential modulation of prothrombotic state by agents affecting LPS requires further testing.

## Introduction

Lipopolysaccharide (LPS) is a structural and protective component of the Gram-negative bacteria outer membrane. Gut microbiota metabolites, including LPS, are detectable in the peripheral circulation as a consequence of the intestinal barrier breakdown. They act as signaling molecules and have been proven to be linked to the cardiovascular health [1, 2]. The gut microbiota composition and intestinal microbial health depend on lifestyle (e.g. dietary intake) and environmental factors (e.g. antibiotic use). Mechanisms linking LPS with the cardiovascular system are multiple. LPS directly interacts with myocardial cells resulting in conduction disturbances (i.e. decreased conduction velocity, triggered ectopic activity, and re-entry) through induced structural and electrical remodeling due to the toll-like receptor 4- and NOD-like receptor protein-3 inflammasome-related inflammatory response [3–5]. In human circulation LPS acts as a pro-aggregating molecule through tissue factor (TF) enhanced expression by monocytes and favoring von Willebrand factor (vWF) endothelial release [4].

It has been demonstrated that aberrant gut microbiota, termed gut dysbiosis, may affect the cardiovascular risk, including that for ischemic stroke [3, 4, 6]. Pastori et al. have found a significant association between circulating LPS and major cardiovascular adverse events in 912 anticoagulated patients with non-valvular atrial fibrillation (AF). In their study patients with serum LPS > 100 pg/mL had the highest risk of myocardial infarction, stroke, coronary revascularization, transient ischemic attack, and cardiovascular death over a median 40-month follow-up (hazard ratio 1.795, 95% CI 1.26–2.52, *p* = 0.001) [7].

The global burden of AF has been progressively increasing, as AF remains the most prevalent sustained cardiac arrhythmia with expected doubling of the number of AF patients by the year 2050 [8, 9]. Oral anticoagulation (OAC) reduces stroke risk by 64% [9]. Despite OAC recommendation in most AF patients [9, 10], a proportion of patients who are not appropriately treated is substantial [11].

Mechanisms of a prothrombotic state in AF patients are complex and still poorly understood [12]. It has been reported that abnormal fibrin clot structure and function, reflected by the formation of more compact networks displaying impaired lysability, reflected by decreased clot permeability (Darcy’s constant, K_s_) and prolonged clot lysis time (CLT) [13, 14], contribute to the increased rates of ischemic cerebrovascular events in patients with AF [15]. Elevated fibrinogen, increased thrombin generation, platelet-derived proteins, and vWF are also involved in unfavorably altered fibrin clot properties, the so-called prothrombotic fibrin clot phenotype [14]. It has also been shown that such prothrombotic fibrin clot features are associated with elevated biomarkers such as cardiac troponin, natriuretic peptides, and growth differentiation factor-15 (GDF-15) in patients with AF [12, 13, 15–17].

To our knowledge, there have been no studies on low-grade LPS-driven endotoxemia as a potential modulator of fibrin clot properties. Given the fact that enhanced inflammation is typically related to higher LPS [2], we hypothesized that elevated LPS concentrations could contribute to prothrombotic fibrin clot characteristics in AF patients. In the present study we aimed to investigate associations of elevated serum LPS on the set coagulation and fibrinolysis markers in patients with AF.

## Methods

### Patients

We prospectively included 157 consecutive anticoagulant-naïve patients with either chronic, or persistent, or paroxysmal AF defined according to the ESC guidelines [18, 19]. The patients were recruited in outpatient clinics in Poland from 2013 to 2018. Patients with any thromboembolism or myocardial infarction within the previous three months, severe co-morbidities such as end-stage kidney disease, liver cirrhosis, malignancy, or signs of acute infection were excluded. We collected data on demographic characteristics, cardiovascular risk factors together with the CHA_2_DS_2_-VASc and HAS-BLED scores, concomitant diseases, and medications [9].

### Laboratory tests

Fasting blood samples were taken from the antecubital vein with minimal stasis. Blood cell counts, glucose, lipid profile, creatinine with estimated glomerular filtration rate, activated partial thromboplastin time (APTT), international normalized ratio (INR), fibrinogen and C-reactive protein (CRP) were assessed.

Calibrated automated thrombography (CAT; Thrombinoscope BV, Maastricht, the Netherlands) in a 96-well plate fluorometer (Ascent Reader, Thermolat Systems OY, Helsinki, Finland) at 37 °C was used to measure endogenous thrombin potential (ETP). Platelet-poor plasma (80 µl) was diluted with a tissue factor (TF)-based activator (20 µl; Diagnostica Stago, Asnières, France) containing recombinant TF (5 pmol l − 1), 4 micromolar phosphatidylserine/ phosphatidylcholine/phosphatidylethanolamine vesicles and FluCa solution (20 µL; Hepes, pH 7.35, 100 nmol/L CaCl_2_, 60 mg/mL bovine albumin and 2.5 mmol/L Z-GlyGly-Arg-amidometylcoumarin).

Clot permeability was assessed following the addition of calcium chloride (20 mmol/L) and human thrombin (1 U/mL, Sigma). Tubes which contained the clots were joined with a reservoir of Tris-buffered saline (0.1 mol/l NaCl, 0.01 mol/l Tris, pH 7.5). The volume flowing for 60 min through the gels was assessed. The permeation coefficient was calculated according to the equation *K*_*s*_=*Q*×*L*×*ƞ*/(*t*×*A* × *Δp*), (Q = the flow rate in time, L = the length of a fibrin gel, ƞ = the viscosity of liquid in poise, t = percolating time, A = the cross sectional area in cm^2^, Δp = a differential pressure in dyne/cm^2^).

Plasma clot lysis time (CLT) was determined using the turbidity method in citrated plasma mixed with calcium chloride (15 mmol/L), human tissue factor (10,000-diluted; Innovin, Siemens) at a final concentration of 0.6 pmol/L, phospholipid vesicles (12 µmol/L) and recombinant tPA (60 ng/mL; Boehringer Ingelheim, Ingelheim, Germany). Assessments were performed at 405 nm at 37 °C. Clot formation was identified as the midpoint of the clear-to-maximum-turbid transition. Therefore, CLT was measured from this time to the midpoint of the maximum turbid-to-clear transition.

Plasma samples (9:1 of 3.2% trisodium citrate) were centrifugated for 20 min at 2500 g and the aliquoted supernatant was stored at − 80 °C until batch analysis. Plasma PAI-1 antigen and t-PA antigen levels were measured by ELISAs (American Diagnostica, Stamford, Connecticut, USA).Thrombin-activatable fibrinolysis inhibitor (TAFI) antigen (Hyphen Biomed). Chromogenic assays to measure plasminogen and α2-antiplasmin activity were used (Siemens, Germany).

N-terminal pro-B-type natriuretic peptide (NT-proBNP) levels were measured using electrochemiluminescence immunoassay (Roche Diagnostics, Mannheim, Germany). GDF-15 was assessed by immunoenzymatic test (ELISA, Quantikine, R&D Systems, Minneapolis, USA). vWF antigen was assessed by an immunoturbidimetric assay (Siemens, Germany).

Serum lipopolysaccharide was determined using a ELISA kit (Cusabio, Wuhan, China) with the detection range from 6.25 to 400 pg/mL.

### Statistical analysis

Continuous data were described by means and standard deviations or medians and interquartile ranges (IQR) for normal and non-normal distributions, respectively. Normality of distribution was checked by Shapiro-Wilk test. Categorical data were summarized by numbers and percentages. Group comparisons were performed using the chi-square or Fisher exact test for categorical variables, and t-test or Mann-Whitney test for normally or non-normally distributed continuous variables, respectively. Due to a non-normal distribution of LPS, Spearman rank correlation coefficients were calculated to assess the strength of monotonic association between LPS and other variables of interest. Logistic regression analysis was performed to determine variables predicting the CLT top quartile. Univariable and multivariable logistic regression models were built and crude odds ratios (OR) with 95% confidence intervals (95% CI) were calculated for the relation between potential predictors and the top quartile of CLT. Predictors were included in this multivariable model if univariable OR was significant with *p*-value < 0.05 and did not shown strong correlation (Spearman rank correlation coefficient > 0.5) with other predictors. For the potential predictors continuous increase multivariable logistic regression models were created by forward selection procedure and then adjusted for age, sex, and fibrinogen. Predictors were included in multivariable models if univariable OR was significant with a *p*-value < 0.05. A two-tailed *p*-value < 0.05 was considered statistically significant. All statistical analyses were performed using the R software package version 4.0.3. Since preliminary data comparing fibrinolysis markers and LPS levels were not available, sample size calculation was based on factor VIII, that has been shown to correlate with LPS increase [20]. Based on preliminary data a sample size of 47 in each group was required to provide 80% power to detect a 20% difference at a two-sided 0.05 level of significance.

## Results

A total of 157 patients (women, 57.3% ) aged from 42 to 86 years, who had a median CHA_2_DS_2_-VASc score of 4.0 (IQR 3.0–5.0), were studied (Table 1). The median AF duration was 5 (2.0–10.0) years. The most common co-morbidities were arterial hypertension (82.2%), dyslipidemia (83.4%), coronary artery disease (46.5%), peripheral artery disease (45.5%), diabetes mellitus (35.7%), and chronic kidney disease (28.0%).

A median LPS was 73.0 (58.0-100.0) pg/mL and ranged from 23.0 pg/mL to 140 pg/mL. There was no association of LPS with age, but females had higher LPS levels (80.0 [63.0-107.5] pg/mL vs. 70.0 [51.2–90.0] pg/mL, *p* = 0.005). The prevalence of co-morbidities and medications used as well as CHA_2_DS_2_-VASc scores were not associated with LPS. Routine laboratory variables including fibrinogen, showed no associations with LPS.

As expected, K_s_ negatively correlated with CLT (*r*=-0.43, *p* < 0.001) and PAI-1 (*r*=-0.23, *p* < 0.001), while CLT positively correlated with ETP (*r* = 0.32, *p* < 0.001) and PAI-1 (*r* = 0.33, *p* < 0.001).

**Fig. 1:**
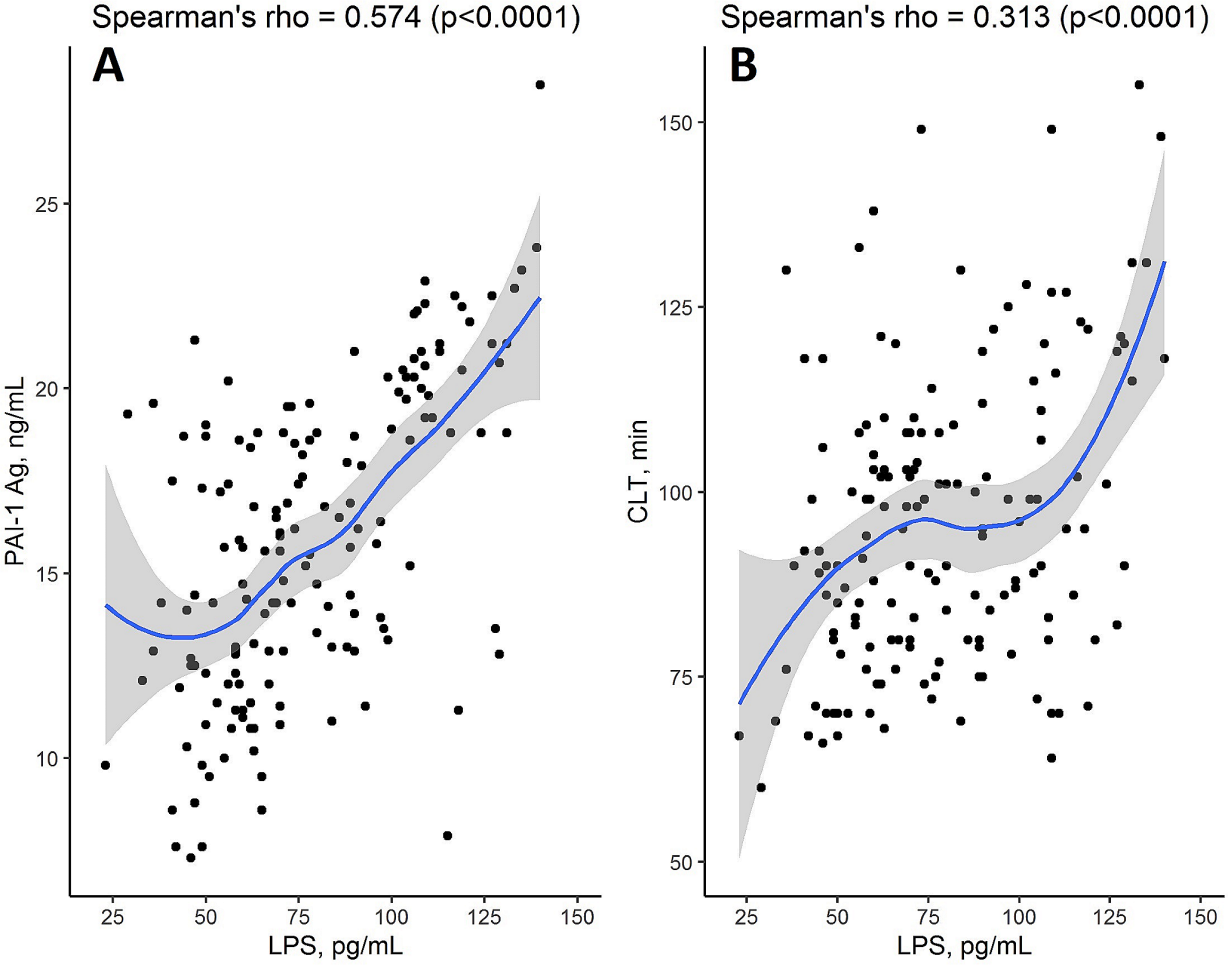
Serum lipopolysaccharide (LPS) Spearman’s correlations with plasminogen activator inhibitor-1 (PAI-1, Panel A) and clot lysis time (CLT, Panel B)

Analysis of fibrin clot properties showed a tendency for an inverse association of LPS with K_s_ (*r*=-0.15, *p* = 0.057), while a significant correlation was observed for CLT (*r* = 0.31, *p* < 0.001) as well as its major determinant, PAI-1 (*r* = 0.57, *p* < 0.001; Fig. 1). No similar associations were found for other fibrinolysis-related proteins such as TAFI (*r*=-0.1, *p* = 0.17), plasminogen (*r* = 0.03, *p* = 0.65), α2-antiplasmin (*r* = 0, *p* = 0.94), and t-PA (*r*=-0.01, *p* = 0.90). However, LPS weakly correlated with vWF (*r* = 0.19, *p* = 0.013). There was no association of LPS and ETP (*r*=-0.03, *p* = 0.68).

In terms of biomarkers, there were associations of LPS with GDF-15 (*r* = 0.27, *p* < 0.001) and cTnI (*r* = 0.16, *p* = 0.045), but not NT-proBNP.

CLT positively correlated with NT-proBNP (*r* = 0.57, *p* < 0.001), cTnI (*r* = 0.27, *p* < 0.001) and GDF-15 (*r* = 0.25, *p* < 0.001). An inverse correlation of K_s_ and NT-proBNP (*r*=-0.29, *p* < 0.001) was noted.

In patients with elevated LPS defined as the top quartile (LPS > 100 pg/mL, *n* = 40) compared to those with LPS < 100 pg/mL, median CLT was prolonged by 16.1% (*p* = 0.001) and median PAI-1 was higher by 45.1% (*p* < 0.001) without any differences in K_s_, other fibrinolysis-related proteins, thrombin generation or vWF. In addition, patients with elevated LPS had GDF-15 higher by 23.8% (Table 2). There were no differences in demographic, clinical and routine laboratory variables between patients with elevated LPS and the remainder (Table 2).

**Table 1:** Patient characteristics

Variable	Total, *n* = 157	Patients with LPS < 100 pg/mL, *n* = 117	Patients with LPS ≥ 100 pg/mL, *n* = 40	*p* value
Age, years	70.0 (65.0–76.0)	70.0 (64.0–75.0)	71.0 (66.0–77.0)	0.37
Female sex	90 (57.3)	73 (62.4)	17 (42.5)	0.028
BMI, kg/m^2^	29.6 (4.8)	29.1 (25.7–33.1)	28.4 (25.4–32.5)	0.49
AF duration, years	5.0 (2.0–10.0)	5.0 (2.0–11.0)	6.5 (3.0–10.0)	0.49
AF type				
chronic	33 (21.0)	25 (21.4)	8 (20.0)	0.85
paroxysmal	78 (49.7)	56 (47.9)	22 (55.0)	0.47
persistent	46 (29.3)	36 (30.8)	10 (25.0)	0.49
CHA_2_DS_2_-VASc	4.0 (3.0–5.0)	4.0 (3.0–5.0)	4.0 (3.0-5.2)	0.81
HAS-BLED	1.0 (1.0–2.0)	1.0 (1.0–2.0)	1.0 (1.0–2.0)	0.41
LVEF, %	47.0 (41.8–55.0)	47.0 (40.0–55.0)	47.2 (42.8–53.1)	0.81
Left atrium, mm	46.0 (42.0–50.0)	45.0 (41.0–50.0)	46.0 (44.0-48.2)	0.34
**Concomitant diseases**				
Arterial hypertension	129 (82.2)	94 (80.3)	35 (87.5)	0.31
Diabetes mellitus	56 (35.7)	45 (38.5)	11 (27.5)	0.21
Dyslipidemia	131 (83.4)	97 (82.9)	34 (85.0)	0.76
Coronary artery disease	73 (46.5)	57 (48.7)	16 (40.0)	0.34
Vascular disease	73 (46.5)	57 (48.7)	16 (40.0)	0.34
CKD	44 (28.0)	31 (26.5)	13 (32.5)	0.47
COPD	14 (8.9)	12 (10.3)	2 (5.0)	0.52
Current smoking	41 (26.1)	31 (26.5)	10 (25.0)	0.85
**Past medical history**				
Prior PCI or CABG	31 (19.7)	20 (17.1)	11 (27.5)	0.43
Prior MI	34 (21.6)	26 (22.2)	8 (20.0)	0.77
Prior stroke or TIA	17 (10.8)	15 (12.8)	2 (5.0)	0.48
Venous thrombosis	17 (10.8)	14 (12.0)	3 (7.5)	0.56
**Medication**				
Beta-blocker	129 (96.2)	96 (82.1)	33 (82.5)	0.95
RAA inhibitors	135 (85.9)	100 (85.5)	35 (87.5)	0.75
CCA	32 (20.4)	22 (18.8)	10 (25.0)	0.4
ASA	61 (38.9)	42 (35.9)	19 (47.5)	0.19
Clopidogrel	9 (5.7)	6 (5.1)	3 (7.5)	0.69
NSAID	36 (22.9)	26 (22.2)	10 (25.0)	0.72
Statin	118 (75.2)	89 (76.1)	29 (72.5)	0.65
Digoxin	27 (17.2)	20 (17.1)	7 (17.5)	0.95
Diuretics	91 (57.9)	69 (59.0)	22 (55.0)	0.66
Insulin	15 (9.6)	12 (10.3)	3 (7.5)	0.76
Oral hypoglycemic drug	32 (20.4)	24 (20.5)	8 (20.0)	0.94
Amiodarone	21 (13.4)	15 (12.8)	6 (15.0)	0.73
Propafenone	12 (7.6)	11 (9.4)	1 (2.5)	0.3

Results are presented as median (interquartile range) or number (percentage). LPS – lipopolysaccharide, BMI – body mass index, AF – atrial fibrillation, CHA_2_DS_2_-VASc and HAS-BLED – stroke risk score and bleeding risk score (see ref. 11), CKD – chronic kidney disease, COPD – chronic obstructive pulmonary disease, PCI – percutaneous coronary intervention, CABG – coronary artery by-pass grafting, MI – myocardial infarction, TIA – transient ischemic attack, RAA – renin-angiotensin-aldosterone, ASA – acetylsalicylic acid, NSAID – non-steroidal anti-inflammatory drug, CCA – calcium channel antagonist.

**Table 2:** Laboratory investigations in atrial fibrillation patients with LPS in the top quartile versus the remainder

Variable	Total, *n* = 157	Patients with LPS < 100 pg/mL, *n* = 117	Patients with LPS≥100 pg/mL, *n* = 40	*p* value
Hemoglobin^*^, g/L	139 (15)	138 (15)	142 (15)	0.40
White blood cells, 10^9^/L	6.5 (5.3–7.4)	6.6 (5.3–7.3)	6.4 (5.5–7.8)	0.51
Platelets, 10^9^/L	199 (165–230)	200 (168–229)	196 (162–236)	0.72
Glucose, mmol/L	5.7 (5.2–6.3)	5.7 (5.2–6.5)	5.6 (5.2–6.2)	0.5
Creatinine, µmol/L	85.4 (73.0-100.0)	87.0 (73.8-100.1)	83.5 (71.7–94.5)	0.24
eGFR^*^, mL/min	70.8 (18.1)	71.4 (18.6)	69.4 (16.7)	0.55
AlAT, U/L	22.0 (18.0–30.0)	22.0 (18.0–31.0)	23.0 (18.0-29.2)	0.61
LDL cholesterol, mmol/L	2.3 (1.9-3.0)	2.2 (1.9-3.0)	2.4 (2.1–2.9)	0.2
TSH, uIU/mL	1.9 (0.9–1.8)	1.8 (1.1–2.9)	1.9 (1.5–2.9)	0.3
CRP, mg/L	1.7 (1.0-2.9)	1.8 (1.0-3.1)	1.6 (1.0-2.2)	0.23
INR^*^	1.0 (0.1)	1.0 (0.1)	1.0 (0.1)	0.63
APTT, s	29.4 (26.5–31.0)	29.4 (26.2–30.9)	29.5 (26.8–32.1)	0.46
NT-proBNP, pg/mL	767 (401–1356)	720 (401–1242)	1138 (404–1628)	0.075
GDF-15, pg/mL	1544.5 (1201.0-1996.5)	1471.5 (1175.2–1880.0)	1821.5 (1418.8–2318.0)	0.005
cTnI, ng/L	6.2 (5.1–7.6)	6.0 (5.1–7.5)	6.9 (5.6–8.3)	0.11
Fibrinogen, g/L	3.2 (2.6–3.9)	3.2 (2.6–3.9)	2.9 (2.3–3.7)	0.051
K_s_, cm^2^ × 10^-9^	6.6 (6.0-7.2)	6.7 (6.1–7.2)	6.5 (5.8-7.0)	0.2
CLT, min	94.0 (80.0-108.0)	90.0 (79.0-103.0)	104.5 (88.2-121.2)	0.001
ETP^*^, nM×min	1456.0 (1388.0-1544.0)	1470.4 (119.2)	1470.7 (143.1)	0.99
vWF^*^, %	196.8 (50.5)	194.2 (50.6)	204.6 (50.3)	0.26
TAFI, %	100 (90–112)	100 (90–113)	98.5 (86.0-108.8)	0.21
plasminogen, %	107 (97–120)	106 (98–117)	110 (96–129)	0.24
α2-antiplasmin, %	106 (96–117)	107 (96–117)	103 (93.2–116.0)	0.58
t-PA Ag, ng/mL	8.9 (6.5–10.9)	8.1 (6.4–11.0)	9.7 (7.4–10.8)	0.74
PAI-1 Ag, ng/mL	15.7 (12.8–19.0)	14.2 (12.0-16.9)	20.6 (19.1–22.0)	< 0.001

The data are shown as mean (standard deviation), or median (interquartile range) as appropriate. Abbreviations: LPS – lipopolysaccharide, eGFR – estimated glomerular filtration rate, AlAT – alanine aminotransferase, L/HDL – low/high density lipoprotein, TG – triglyceride, TSH – thyroid-stimulating hormone, INR – international normalized ratio, APTT – activated partial thromboplastin time, CRP – C-reactive protein, NT-proBNP – N-terminal pro-B-type natriuretic peptide, K_s_ – permeation coefficient, CLT – clot lysis time, ETP – endogenous thrombin potential, TAFI, thrombin-activatable fibrinolysis inhibitor, t-PA – tissue plasminogen activator, PAI-1 – plasminogen activator inhibitor, GDF – growth differentiation factor, cTn – cardiac troponin, Ag – antigen

Patients with hypofibrinolysis, defined as the top quartile of CLT (*n* = 42, CLT≥108 min, female sex 59.5%, median CHA_2_DS_2_-VASc score 4.0 [3.0–5.0]) had LPS greater than the remaining patients (91.5 pg/mL [69.2–116.0] vs. 70.0 pg/mL [55.0–90.0], *p* < 0.001). Similar analysis for K_s_ showed no difference in LPS levels between patients with decreased clot permeability characterized by K_s_ below the top quartile (*n* = 115, K_s_<7.2 cm^2^ × 10^-9^) and the remaining group (76.0 pg/mL [60.0-103.5] vs. 68 pg/mL [50.0–90.0], *p* = 0.26).

The association of PAI-1 and LPS was the strongest of all variables measured. Patients with PAI-1 in the top quartile (*n* = 40, PAI-1 Ag≥19 ng/mL) had LPS greater than their counterparts (67.0 pg/mL [56.0–84.0] vs. 108.5 pg/mL [101.2–119.0], *p* < 0.001).

In multivariable logistic regression analysis a two-way approach was adopted (i.e. a predictor in the top quartile vs. predictor as a continuous variable) to infer a casual relationship between CLT and the predictors. LPS was the independent predictor of prolonged CLT defined as the top quartile in both models (Model #1 and #2, Table 3) only if PAI-1 was excluded from the univariable models. The PAI-1 inclusion resulted in LPS elimination from the multivariable model by the forward selection procedure and thus rendered PAI-1 the strongest predictor of CLT (Model #3, Table 3).

**Table 3:** Uni- and multivariable logistic regression models with potential predictors of prolonged clot lysis time (the top quartile, CLT ≥ 108 min; *n* = 42)

Variable	uni-variable OR	95% CI	*p* value	multi-variable OR	95% CI	*p* value
**Model #1: Potential predictor in the top quartile**						
LPS ≥ 100 pg/mL	3.7	1.71–7.99	< 0.001	3.36	1.5–7.52	0.003
vWF ≥ 227%	2.33	1.08–5.03	0.03	2.21	0.98-5	0.057
GDF-15 ≥ 1996 pg/mL	2.43	1.12–5.27	0.024	1.67	0.73–3.85	0.23
cTnI ≥ 7.63 ng/L	1.52	0.69–3.33	0.299			
**Model #2: Potential predictor as a continuous variable**						
LPS, pg/mL	1.03	1.01–1.04	< 0.001	1.03^*^	1.01–1.05	0.003
cTnI, ng/L	1.36	1.13–1.65	0.001	1.33^*^	1.02–1.73	0.035
ETP, per 10 nM×min	1.04	1.01–1.07	0.012	0.97^*^	0.92–1.02	0.2
NT-proBNP, per 100 pg/mL	1.16	1.1–1.24	< 0.001	1.2^*^	1.1–1.31	< 0.001
**Model #3: Potential predictor as a continuous variable (PAI-1 included)**						
LPS, pg/mL	1.03	1.01–1.04	< 0.001			
cTnI, ng/L	1.36	1.13–1.65	0.001	1.29^*^	0.99–1.68	0.054
ETP, per 10 nM×min	1.04	1.01–1.07	0.012	0.95^*^	0.91–1.01	0.08
NT-proBNP, per 100 pg/mL	1.16	1.1–1.24	< 0.001	1.22^*^	1.11–1.34	< 0.001
PAI-1 Ag, ng/mL	1.25	1.12–1.38	< 0.001	1.34^*^	1.16–1.55	< 0.001

* adjusted for age, sex, and fibrinogen

Abbreviations see Table 2

## Discussion

In the present study we are first to demonstrate that anticoagulant-naïve patients with AF and elevated serum lipopolysaccharide have the prothrombotic fibrin clot phenotype characterized mainly by decreased susceptibility of fibrin clots to tPA-mediated lysis. High LPS levels were associated not only with impaired fibrinolysis, as reflected by a global fibrinolysis assessment, i.e. prolonged CLT in association with increased PAI-1, but also endothelial injury, reflected by increased vWF, along with oxidative stress and inflammation, reflected by elevated GDF-15. Importantly, elevated LPS was the independent predictor of prolonged CLT in AF. Our findings shed new light on prothrombotic effects of low-grade endotoxemia in humans, indicating that in AF and possibly in other cardiovascular diseases [21], elevated LPS impairs fibrinolysis largely via increased PAI-1 concentrations in circulating blood, which might at least in part explain an increased risk of ischemic cardiovascular events in nonseptic patients with elevated LPS. We also observed CLT in association with NT-proBNP and cTnI, which is in line with the previous findings and supports their utility in the multi-marker assessment of the prothrombotic state in patients with AF [22–24].

The conversion of plasminogen into plasmin by t-PA is under control of PAI-1 released from platelets, endothelium, hepatocytes, adipocytes, and, to some extent, from fibroblasts [14, 25]. Several antifibrinolytic proteins (i.e. PAI-2, α2-antiplasmin, TAFI) further contribute to fibrinolysis efficiency in vivo [14]. Enhanced PAI-1 release in response to inflammation has been demonstrated to be associated with structural and electrical remodeling of the atria in patients with AF [26]. In experimental models overproduction of PAI-1 by endothelial cells via TLR4-related inflammatory response to LPS addition has been observed [2]. As PAI-1 acts as an acute phase protein, it is secreted by a number of cells in response to inflammatory cytokines [25, 27]. It is postulated that the LPS-induced PAI-1 expression is modulated through NF-κB and MAP kinases activation [2, 25]. Consequently, LPS induces PAI-1 oversecretion and thus, initiates an antifibrinolytic response [28, 29]. These molecular pathways might contribute to the antifibrinolytic effects in the setting of low-grade LPS-related endotoxemia. Our findings suggest that LPS is potent enough to modulate fibrinolysis in human circulation, however the underlying mechanisms remain unclear and require further research.

Prothrombotic clot phenotype and its role in the prediction of thrombotic and bleeding complications have been reported in AF patients regardless of the CHA_2_DS_2_-VASc score [15, 17, 22, 30]. Impaired fibrinolysis in AF patients reflected by elevated plasma PAI-1 is associated with thromboembolic events [31]. However, little is known about fibrin clot properties modulation by enhanced LPS levels. To the best of our knowledge, the only study on this topic was published by Nunes et al. [32], who investigated the effects of LPS on clot formation and architecture in plasma from healthy individuals and in purified fibrinogen models. They found the LPS-dependent formation of denser fibers and altered clot mechanics, but they did not evaluate the clot permeability, which is a well-established indirect measure of clot density, and clot lysability [32]. Although K_s_ has been demonstrated to be lower in cardiovascular disorders associated with increased risk of thromboembolism, including AF [14], in our patients LPS showed no significant impact on this variable. Taken together, higher LPS modulates efficiency of fibrinolysis, which increases the current knowledge on the complex regulation of this process in AF and possibly in other cardiovascular diseases.

We also found that LPS is weakly positively associated with vWF antigen. In the study by Carnevale et al. LPS strongly correlated with vWF in liver cirrhosis patients, which was attributed to the LPS-related endothelial release of factor VIII and vWF [20]. Despite vWF is being primarily considered a marker of endothelial cells injury, it is also synthetized and stored in platelets. It is known that vWF is increased in AF [33]. It might be speculated that LPS exerts the native impact on prognosis in part by vWF mediated effects.

Menichelli et al. reported unfavorable antioxidant status in AF patients with elevated circulating LPS [34]. Hu et al. linked the thrombus formation with elevated GDF-15 in anticoagulant-naïve AF patients [35]. In our study, a novel findings is the association of LPS and GDF-15, which may reflect inflammatory actions of LPS in concordance with other findings [2, 25, 27]. It might be hypothesized that enhanced GDF-15 level, currently known as an integrative disease severity marker [36], might in part contribute to prothrombotic effects of LPS in AF [24].

Altered fibrin clot properties in AF patients can predict cerebrovascular outcomes [30, 37].

Impaired fibrinolysis in AF patients reflected by elevated PAI-1 is associated with thromboembolic events [31]. cTnI and vWF, markers of myocardial and endothelial injury, have also been reported to predict clinical outcomes in patients with AF [38–40]. The biomarker substudy in the ARISTOTLE trial with apixaban has demonstrated that GDF-15 alone and in addition to cTnI are predictive of major bleeding, mortality, and stroke in AF patients [36]. We found a significant association between elevated LPS and cTnI, vWF, and GDF-15. It would be interesting to investigate cerebrovascular outcomes in relation to the low-grade LPS-related endotoxemia, however this issue was beyond the scope of our study.

A potential impact of concomitant medications deserves a comment. Since there is evidence that aspirin, statins, antihypertensive medication, and antidiabetic therapy, may improve both K_s_ and CLT [14, 41], in our study such effects were not related to LPS. In the context of anticoagulation which potently affects fibrin clot properties [14], it should be highlighted that our study group involved solely AF patients who were not treated with any anticoagulant at the enrollment. To our knowledge, there have been no reports showing that anticoagulation affects low-grade endotoxemia, therefore it might be speculated that benefits from anticoagulation, including less prothrombotic fibrin clot phenotype, are not mediated by decrease in circulating LPS concentrations. We consider this the strength of our study, as the current OAC prescription rates reach 87% at one year from the therapy initiation [42].

### Study limitations

Although the sample size was relatively small, it was sufficiently powered to show intergroup differences. The study has inherent limitations of a cross-sectional design. We did not assess the dietary intake. No other markers of dysbiosis including short-chain fatty acids, bile acids, and trimethylamine N-oxide were measured. The results cannot be extrapolated to the patient subsets defined in the exclusion criteria and those treated with OAC [43]. All laboratory findings were assessed once therefore some changes with time (i.e. the management strategy including medication) cannot be excluded given the inclusion period over a time span of five years. The fibrin clot-related measurements are feasible, however further work on the standardization is warranted [13], and now CLT cannot be used in everyday practice. Finally, mechanistic studies to explore precise mechanisms behind PAI-1-associated hypofibrinolysis in subjects with elevated LPS were beyond scope of the present research, and the study should be considered hypothesis-generating.

In conclusion, elevated serum LPS was the independent predictor of prolonged CLT in anticoagulant-naïve patients with AF. The antifibrinolytic effect was largely driven by enhanced PAI-1 release. Further studies are needed to elucidate the impact of low-grade endotoxemia on a prothrombotic state in AF patients and its potential modulation by agents affecting LPS in this clinical context.
